# *Flagellimonas algicida* sp. Nov.: A Novel Broad-Spectrum Algicidal Bacterium Targeting Harmful Algal Bloom Species and Genomic Insights into Its Secondary Metabolites

**DOI:** 10.3390/microorganisms13092062

**Published:** 2025-09-04

**Authors:** Ning Wang, Yiling Liang, Hui Zhou, Yutian Chi, Lizhu Chen, Qiliang Lai, Hong Xu

**Affiliations:** 1 State Key Laboratory of Cellular Stress Biology, Key Laboratory of the Ministry of Education for Coastal and Wetland Ecosystems, School of Life Sciences, Xiamen University, Xiamen 361102, China; wn2020080045@126.com (N.W.); ncuskliangyiling@163.com (Y.L.); 21620231153551@stu.xmu.edu.cn (H.Z.); 21620231153555@stu.xmu.edu.cn (Y.C.); clz0823@xmu.edu.cn (L.C.); 2 Key Laboratory of Marine Biogenetic Resources, Third Institute of Oceanography, Ministry of Natural Resources, Xiamen 361005, China; lai719@163.com

**Keywords:** *Flagellimonas algicida* sp. nov., harmful algal bloom, biocontrol, polyphasic taxonomy, *Phaeocystis globosa* colony

## Abstract

A novel Gram-negative bacterium, designated strain SN16^T^, was isolated from a harmful algal bloom (HAB). Strain SN16^T^ exhibited potent, broad-spectrum algicidal activity against the colony-forming alga *Phaeocystis globosa* and eight other HAB-causing species, highlighting its potential as a promising candidate for the biological control of HABs. A phylogenetic analysis of 16S rRNA gene sequences placed strain SN16^T^ within the genus *Flagellimonas*. The average nucleotide identity (ANI) and digital DNA–DNA hybridization (dDDH) values between strain SN16^T^ and its relatives were 75.4–91.4% and 19.3–44.0%, respectively. These values fall below the established thresholds for species delineation, confirming that SN16^T^ represents a novel species. A chemotaxonomic analysis revealed its dominant cellular fatty acids to be iso-C_15:0_ and iso-C_15:1_ G. The major polar lipid was phosphatidylethanolamine, and the primary respiratory quinone was menaquinone-6. Genome mining identified 11 biosynthetic gene clusters (BGCs), including those encoding for terpenes, ribosomal peptide synthetases, and non-ribosomal peptide synthetases. By integrating BGC analysis with the observed algicidal activities, we predicted that pentalenolactone and xiamycin analogues are the likely causative compounds. Based on this polyphasic evidence, strain SN16^T^ is proposed as a novel species of the genus *Flagellimonas*, named *Flagellimonas algicida* sp. nov. This is the first report of *Flagellimonas* species exhibiting broad-spectrum algicidal activity, including activity against the colonial form of *P. globosa*—a key ecological challenge in HAB mitigation.

## 1. Introduction

Over the past decade, blooms of the haptophyte *Phaeocystis globosa* Scherffel have become increasingly frequent in the East and South China Seas, significantly impacting the coastal environment, disrupting aquaculture, and posing risks to human health [1,2]. *P. globosa* exhibits a complex, polymorphic life cycle with two primary forms: small (3–10 μm), biflagellate, free-living solitary cells and large colonies composed of thousands of cells embedded within a gelatinous, mucopolysaccharide matrix. These colonies, which can range from 100 μm to 3 cm in diameter, are the dominant morphotype during bloom events. The accumulation and subsequent mass decay of these gelatinous colonies harm farmed fish and shellfish through two primary mechanisms: physical obstruction of gills and the release of hemolytic toxins. This process also leads to substantial foam generation, oxygen depletion, and widespread environmental degradation [3,4]. Furthermore, large colonies can pose a significant threat to coastal nuclear power plants by obstructing the filter screens of cooling water intake systems [5].

The profound ecological and economic impacts of *P. globosa* blooms have driven considerable research into mitigation strategies. Physical and chemical methods, including modified clay [6], enhanced cellulose nanocrystal coagulation [7], and polyvinylpyrrolidone-modified pyrite activated persulfate (PVP-FeS2/PS) [8], have been explored in this context. However, their effectiveness is often limited, and concerns such as secondary pollution, high costs, physical damage to aquatic ecosystem, and impracticality hinder their larger-scale application. Microbial control has emerged as a promising alternative, valued for its high efficiency and environmentally friendliness in managing harmful algal blooms. Among microbial agents, algicidal bacteria are particularly promising due to their rapid reproduction, high efficiency, and potential for host specificity [9]. Consequently, numerous bacterial strains with potent algicidal activity against *P. globosa* have been identified, including species from the genera *Bacillus* [10,11], *Streptomyces* [12,13,14,15], *Microbacterium* [16], *Hahella* [17], and *Microbulbifer* [18]. However, a significant limitation is that these bacterial strains primarily target free-living solitary cells and exhibit limited efficacy against the more resilient colonial morphotype. Therefore, isolating novel bacteria with specific algicidal activity against the dominant colonial morphotype remains a critical research objective.

The genus *Flagellimonas*, a member of the family *Flavobacteriaceae*, was first proposed by Bae et al. [19] and subsequently emended by Yoon and Oh [20], Choi et al. [21], and Novoa et al. [22]. A recent taxonomic revision reclassified some species from the genera *Allomuricauda* and *Muricauda* into *Flagellimonas* [22], expanding the genus to 45 species with valid published names (https://lpsn.dsmz.de/genus/flagellimonas, accessed on 15 July 2025) [23]. Members of this genus are typically Gram-stain-negative, non-motile, strictly or facultatively aerobic, yellow-pigmented rods. Their DNA G + C contents range from 41.0 to 55.0 mol%. *Flagellimonas* species are frequently isolated from diverse saline environments, including intertidal zones, salt lakes, seawater, marine sediment, sponges, shrimp gills, the rhizosphere of marine macroalgae, and the phycospheres of dinoflagellates [20,24,25,26,27,28,29,30,31,32,33,34,35]. Despite the frequent discovery of new species, the ecological roles and biotechnological potential of *Flagellimonas* remain largely unexplored. Nevertheless, a few studies have hinted at their potential, revealing capabilities such as the production of the antioxidant zeaxanthin by *F. aquimarina* JCM 11811^T^, *F. flavescens* JCM 11812^T^, *F. lutimaris* KCTC 22173^T^, *F. lutaonensis* KCTC 22339^T^, and *F. olearia* JCM 15563^T^ [36,37,38]; the degradation of *N*-acyl homoserine lactones (AHLs) involved in quorum sensing by *F. olearia* Th120^T^ [39,40,41]; and the enzymatic breakdown of fucoidan by *F. eckloniae* [42]. To the best of our knowledge, however, the algicidal capabilities of this genus have not yet been reported. Therefore, we aimed to isolate and characterize a novel algicidal bacterium with activity against both solitary and colonial forms of *P. globosa*, determine its taxonomic position, and evaluate its genomic potential for secondary metabolite production (Figure S1).

In this study, we isolated a novel bacterial strain, designated SN16^T^, from a seawater sample collected during a *P. globosa* bloom in the East China Sea. This strain exhibits potent algicidal activity against both solitary cells and, most importantly, the colonial morphotype of *P. globosa*. A polyphasic taxonomic approach revealed that strain SN16^T^ represents a novel species within the genus *Flagellimonas*. We further demonstrated that this strain possesses broad-spectrum algicidal activities against eight other HAB-causing species and report, for the first time, the algicidal capabilities of three additional *Flagellimonas* species. This finding suggests that algicidal activity may be a more widespread trait within this genus than previously recognized. This is the first report of *Flagellimonas* species exhibiting broad-spectrum algicidal activity, including activity against the colonial form of *P. globosa*—a key ecological challenge in HAB mitigation.

## 2. Materials and Methods

### 2.1. Bacterial Strains and Culture Conditions

Strain SN16^T^ was isolated in December 2023 from a surface seawater sample collected during a *P. globosa* bloom off Dadeng Island, Xiamen, China (24°30′56.28″ N, 118°19′43.08″ E). Single colonies and pure cultures of strain SN16^T^ were obtained through serial 10-fold dilution and repeated four-zone streaking on ZoBell 2216E agar plates. Strain SN16^T^ was cultured at 30 °C and preserved long-term as a suspension in ZoBell 2216E medium supplemented with 20% (*w*/*v*) glycerol at −80 °C. The novel strain SN16^T^ has been deposited at the Marine Culture Collection of China (MCCC, Xiamen, China) under the accession number MCCC 1K09924^T^ and at the Korean Collection for Type Cultures (KCTC, Jeongeup-si, Jeollabuk-do, Republic of Korea) under the accession number KCTC 102450^T^. Reference strains *F. alvinocaridis* SCR12^T^, *F. crocea* DH64^T^, and *F. chongwuensis* HICW^T^ were obtained from the MCCC. All bacterial strains were routinely cultured in ZoBell 2216E broth at 30 °C with shaking.

### 2.2. Algal Strains and Culture Conditions

The sixteen algal species used in this study were obtained from the Culture Collection Center of Marine Algae (CCMA), State Key Laboratory of Marine Environmental Science (MEL) at Xiamen University, China: *Chlorella vulgaris* Beijerinck, *Tetraselmis helgolandica* (Kylin) Butcher, *Nannochloris oculata* (Droop) D. J. Hibberd, *Prorocentrum donghaiense* D. Lu, *Phaeocystis globosa* Scherffel, *Amphidinium carterae* Hulburt, *Alexandrium tamarense* (Lebour) Balech, *Alexandrium catenella* (Whedon & Kofoid) Balech, *Karenia mikimotoi* (Miyake & Kominami ex Oda) G. Hansen & Moestrup, *Scrippsiella trochoidea* (F.Stein) A. R. Loeblich, *Entomoneis alata* (Ehrenberg) Ehrenberg, *Conticribra weissflogii* (Grunow) K.Stachura-Suchoples & D.M.Williams, *Skeletonema costatum* (Greville) Cleve, *Cylindrotheca closterium* (Ehrenberg) Reimann & J. C. Lewin, *Isochrysis galbana* Parke, and *Heterosigma akashiwo* (Hada) Hada ex Y. Hara & Chihara. Algal cultures were maintained in sterile f/2 medium prepared with natural seawater at 20 °C under a 12:12 h light/dark cycle with a light intensity of 60~100 μmol·photons·m^−2^·s^−2^. Algal cell density was monitored using a Countstar IC1000 automated cell counter (ALT Life Science, Shanghai, China). For all experiments, algal cultures were harvested during the exponential growth phase.

### 2.3. Analysis of Algicidal Characteristics of Strain SN16^T^

To characterize the relationship between bacterial growth and algicidal activity, strain SN16^T^ was cultured in 100 mL of ZoBell 2216E broth at 30 °C with shaking at 200 rpm. Bacterial growth was monitored by measuring the optical density at 600 nm (OD_600nm_). For the algicidal activity analysis, aliquots of the bacterial culture were collected every three hours and added to cultures of *P. globosa* at a final concentration of 10% (*v*/*v*). A culture of *P. globosa* supplemented with 10% 2216E medium served as the negative control. Algal cell densities were determined at specified time points using a Countstar automated cell counter. The algicidal activity was calculated using the following formula:

Algicidal activity (%) = (C_0_ − C_t_)/C_0_ × 100 (1)

where C_0_ and C_t_ represent the cell density at the beginning and at time (t) of the treatment, respectively [43]. All experiments were performed in triplicate.

### 2.4. Determination of Algicidal Mode

To determine whether the algicidal activity was mediated by direct cell contact or secreted compounds, strain SN16^T^ was cultured to the stationary phase. The culture was then fractionated via centrifugation (6000 rpm, 8 min) and subsequent filtration of the supernatant through a sterile 0.22 μm membrane was performed to obtain a cell-free supernatant. The resulting bacterial cell pellet was washed and resuspended in sterile f/2 medium to its original volume. The following treatments were added to separate cultures of *P. globosa* at a final concentration of 10% (*v*/*v*): the whole bacterial culture, the cell-free supernatant, and the resuspended bacterial cells. A culture supplemented with 10% (*v*/*v*) 221E medium was used as the negative control. The treated cultures were incubated at 20 °C under a 12:12 h light/dark cycle. Algicidal activity was measured after 6 and 12 h. All treatments were performed with three biological replicates.

### 2.5. Dose-Dependent Algicidal Activity Against P. globosa Morphotypes

The algicidal efficacy of the SN16^T^ cell-free supernatant against the two primary morphotypes of *P. globosa* was assessed. The supernatant was added to cultures of solitary cells (initial density of 3.34 × 10^6^ cells/mL) and colonial cultures (initial density of 10 colonies/mL) to achieve final concentrations of 1%, 3%, 5%, 7%, and 10% (*v*/*v*). Algicidal activity was calculated after a defined incubation period. Changes in cell morphology and chlorophyll autofluorescence were observed using an inverted fluorescence microscope (Olympus IX71, Olympus, Tokyo, Japan).

### 2.6. Algicidal Spectrum of Strain SN16^T^

To investigate the host specificity of strain SN16^T^, the 16 algal species listed above—representing the phyla Chlorophyta, Pyrrophyto, Bacillariophyta, Chrysophyta, Haptophyta, and Xanthophyta—were selected for an algicidal spectrum analysis. The SN16^T^ cell-free supernatant was added to each algal culture to a final concentration of 10% (*v*/*v*). A corresponding negative control containing 10% 2216E medium was included for each algal species. Algicidal activity was measured after treatment for 24 h. All treatments were conducted in triplicate.

### 2.7. Polyphasic Taxonomic Characterization of Strain SN16^T^

To determine the taxonomic position of strain SN16^T^, a polyphasic approach combining phylogenetic, genomic, phenotypic, physiological, and chemotaxonomic analyses was employed.

#### 2.7.1. Phylogenetic and Genomic Analysis

The 16S rRNA gene was amplified using the universal primers 27F (5′-AGAGTTTGATCMTGGCTCAG-3′) and 1492R (5′-TACGGYTACCTTGTTACGAACTT-3′), as previously described [44]. The resulting PCR amplicon was sequenced by Sangon Biotech. Co., Ltd. (Shanghai, China). The 16S rRNA gene sequence of strain SN16T has been deposited in the GenBank database under the accession number PQ877289. Sequence similarity searches were conducted using the EzBioCloud database ver.2025.04.21 (https://www.ezbiocloud.net/, accessed on 4 July 2025) [45] and the NCBI BLAST N 2.17.0+ (https://www.ncbi.nlm.nih.gov/, accessed on 4 July 2025) [46]. For the phylogenetic analysis, multiple sequence alignments were performed using the CLUSTAL-W algorithm within the MEGA v11.0.13 software [47]. Phylogenetic trees were constructed using the maximum likelihood (ML), neighbor-joining (NJ), and minimum evolution (ME) algorithms. Evolutionary distances were computed using the Kimura 2-parameter model [48], and the topological robustness of the trees was evaluated via bootstrap analysis with 1000 replicates.

For the whole-genome analysis, genomic DNA was extracted using a commercial kit according to the manufacturer’s protocol and stored at −20 °C. Genome sequencing was conducted on an Illumina NovaSeq platform by Majorbio Bio-pharm Technology Co., Ltd. (Shanghai, China). The genome was assembly de novo using SOAPdenovo v2.04 (https://github.com/aquaskyline/SOAPdenovo2, accessed on 5 January 2025) [49] with multiple-Kmer parameters [50]. The DNA G + C content and sequencing depth were calculated using Bowtie2 v2.5.1 (http://bowtie-bio.sourceforge.net/bowtie2/index.shtml, accessed on 5 January 2025) [51]. The draft genome sequence of strain SN16^T^ has been deposited in the GenBank database under the accession number JBMYIX000000000. Protein-coding sequences (CDSs) were predicted using Prodigal v2.6.3 [52] and functionally annotated against the GenBank database. Pairwise digital DNA–DNA hybridization (dDDH) values were calculated using the Genome-to-Genome Distance Calculator 3.0 (http://ggdc.dsmz.de/ggdc.php, accessed on 17 January 2025) [53]. Average nucleotide identity (ANI) values were calculated using the OrthoANIu v1.0 algorithm via the EzbioCloud online tool (https://www.ezbiocloud.net/tools/ani, accessed on 17 January 2025) [54]. A whole-genome-based phylogenetic tree was constructed using KBase (https://www.kbase.us/, accessed on 18 February 2025) [55]. Potential secondary metabolite biosynthetic gene clusters (BGCs) were identified using antiSMASH v8.0.1 (https://antismash.secondarymetabolites.org/#!/start, accessed on 10 July 2025) [56,57]. To further clarify the phylogenetic and evolutionary relationships, the genomes of 45 strains showing the highest 16s rRNA gene similarities to strain SN16^T^ were obtained from public databases.

#### 2.7.2. Phenotypic, Physiological, and Biochemical Characterization

The colony morphology of strain SN16^T^ was observed on ZoBell 2216E agar plates after 2 days of incubation at 30 °C under aerobic conditions. Cell morphology was examined using a scanning electron microscope (JSM-6390, JEOL Co., Tokyo, Japan). Gram staining was performed using a commercial kit (Hunan BKMAM Holding Co., Ltd., Changsha, China) following the manufacturer’s instructions. Catalase activity was assessed through the formation of bubbles after adding a drop of 3% (*v*/*v*) H_2_O_2_ to a fresh colony. Oxidase activity was determined using a commercial reagent (Shanghai Yuanye Bio-Technology Co., Ltd., Shanghai, China).

The growth conditions of strain SN16^T^ were determined as follows. The temperature range for growth was assessed on 2216E agar at various temperatures (4, 15, 20, 25, 30, 37, 40, and 45 °C). The pH range for growth (pH 3.0–10.0, adjusted with 6 M NaOH and 6 M HCl) was determined by monitoring OD_600_ in 2216E broth at 30 °C over 7 days. NaCl tolerance was evaluated by measuring OD_600_ in ZoBell 2216E broth supplemented with NaCl concentrations ranging from 0 to 13% (*w*/*v*, in 1% increments).

Biochemical characteristics, including enzyme activities and carbon source utilization, were investigated using API 20E, API 20NE, and API ZYM kits (bioMérieux, Marcy-l'Étoile, France) [58] following the manufacturer’s instructions. Antibiotic susceptibility was tested using the disk diffusion method on 2216E agar. The following antibiotic discs (Hunan BKMAM Holding Co., Ltd.) were used (µg per disc): chloramphenicol (30), penicillin (10 U), erythromycin (15), neomycin (30), gentamicin (10), kanamycin (30), ampicillin (20), cefazoline (30), cefoperazone (75), streptomycin (10), vancomycin (30), and novobiocin (30).

#### 2.7.3. Chemotaxonomic Characterization

For the analysis of cellular fatty acids, polar lipids, and isoprenoid quinones, the strain was cultured in 2216E broth for 24 h at 30 °C, and the cells were harvested via centrifugation (6000 rpm, 4 °C). Cellular fatty acid methyl esters (FAMEs) were prepared and analyzed using Sherlock’s microbial identification system (MIDI, version 6.0B). Polar lipids were extracted and identified via two-dimensional thin-layer chromatography (TLC), as described by Minnikin et al. [59]. Isoprenoid quinones were extracted and analyzed via HPLC-MS (Waters Corporation, Milford, MA, USA) following the method described by Huang et al. [60].

### 2.8. Statistical Analysis

All experiments were conducted in triplicate, and the results are reported as the mean ± SD. All data analyses were performed using the GraphPad Prism software (version 9.0). Multiple comparisons were conducted, and the Bonferroni method was used to correct the statistical significance, with *p* < 0.05 (*), *p* < 0.01 (**), *p* < 0.001 (***), and *p* < 0.0001 (****).

## 3. Results

### 3.1. Growth and Algicidal Characteristics of Strain SN16^T^

**Figure 1:**
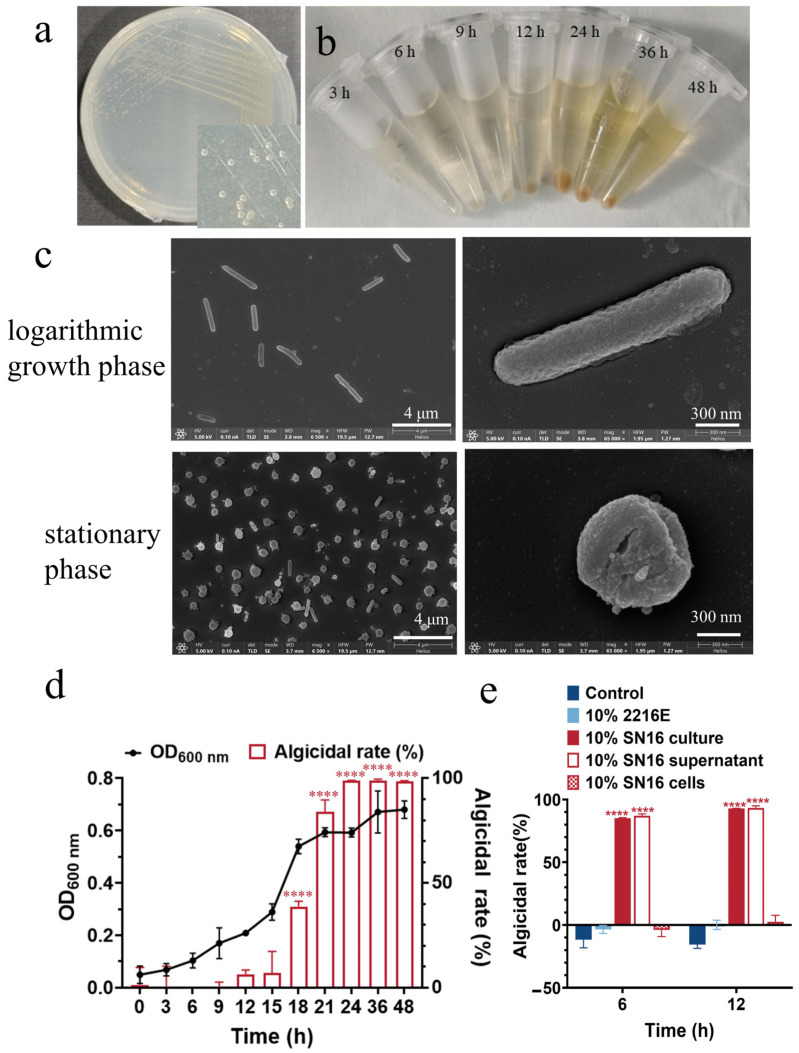
Morphology of strain SN16^T^ and its algicidal activity against *Phaeocystis globosa*. (**a**) Colonies on a 2216E agar plate after 48 h of incubation. (**b**) Cultures in 2216E broth at different growth phases. (**c**) Cell morphological observation at different growth phases using an SEM. (**d**) Growth curve and algicidal activity against *P. globosa*. (**e**) Analysis of algicidal mode. **** indicates the statistical significance (*p* < 0.0001).

Strain SN16^T^ forms round, light-yellow colonies with smooth surfaces and regular edges on 2216E agar plates. The strain exhibited morphological changes dependent on its growth phase. During the logarithmic growth phase, the cells were non-flagellated, long rods, with an average length of 1.84 ± 0.714 μm and a width of 0.34 ± 0.04 μm. Upon entering the stationary phase, cells folded and twisted into a coccoid shape with an average diameter of 0.58 ± 0.13 μm (Figure 1a–c). Algicidal activity of strain SN16^T^ was coupled with its growth cycle. Activity first appeared during the mid-logarithmic phase and then increased progressively. The maximum algicidal rate of 95% against *P. globosa* was achieved after 21 h of cultivation, coinciding with the strain’s entry into the stationary phase (Figure 1d).

To elucidate the algicidal mode, *P. globosa* was treated with either the whole bacterial culture, the cell-free supernatant, or the resuspended bacterial cells. Both the whole culture and cell-free supernatant exhibited strong algicidal effects, achieving removal rates of 93.2% and 92.9%, respectively, after 12 h of co-incubation. In contrast, the resuspended bacterial cells showed negligible activity (Figure 1e). These results indicate that strain SN16^T^ executes its algicidal function primarily through the secretion of extracellular bioactive compounds, characteristic of an indirect attack mode.

### 3.2. Dose-Dependent Algicidal Effects on P. globosa Morphotypes

**Figure 2:**
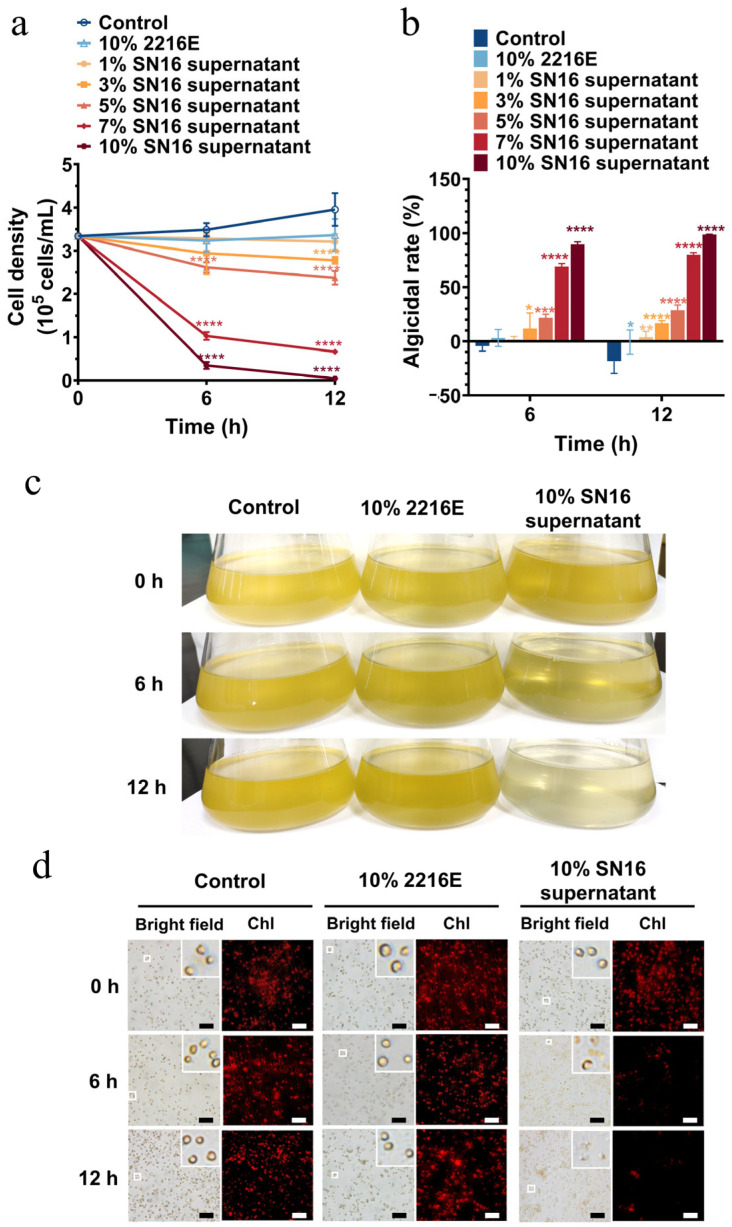
Algicidal activity of cell-free supernatant from strain SN16^T^ against solitary cells of *Pheaocystis globosa*. (**a**) Algicidal activities at various supernatant concentrations (*v*/*v*). The asterisk indicates the statistical significance, with *p* < 0.05 (*), *p* < 0.01 (**), *p* < 0.001 (***), and *p* < 0.0001 (****). (**b**) Corresponding algicidal rates calculated after 6 h and 12 h of co-incubation. (**c**) Algal cultures treated with the 10% supernatant. The asterisk indicates statistical significance, with *p* < 0.05 (*), *p* < 0.01 (**), *p* < 0.001 (***), and *p* < 0.0001 (****). (**d**) Changes in chlorophyll autofluorescence in algal cells after treatment with the 10% supernatant. Bar = 40 μm. Chl, chlorophyll.

The algicidal efficacy of the SN16^T^ cell-free supernatant against both solitary cells and the colonial form of *P. globosa* was evaluated. The activity against solitary cells was strongly dose-dependent (Figure 2a,b). The supernatants at concentrations of 1% and 3% (*v*/*v*) exerted minimal effects, whereas concentrations of 5% and higher induced significant algal cell death. After 12 h of treatment, the 7% and 10% supernatant treatments resulted in algicidal rates of 80.2% and 98.6%, respectively. Treatment with the 10% supernatant caused the algal culture to lighten in color by 6 h and become clear by 12 h, which corresponded to a marked reduction in both cell density and chlorophyll fluorescence (Figure 2c,d).

**Figure 3:**
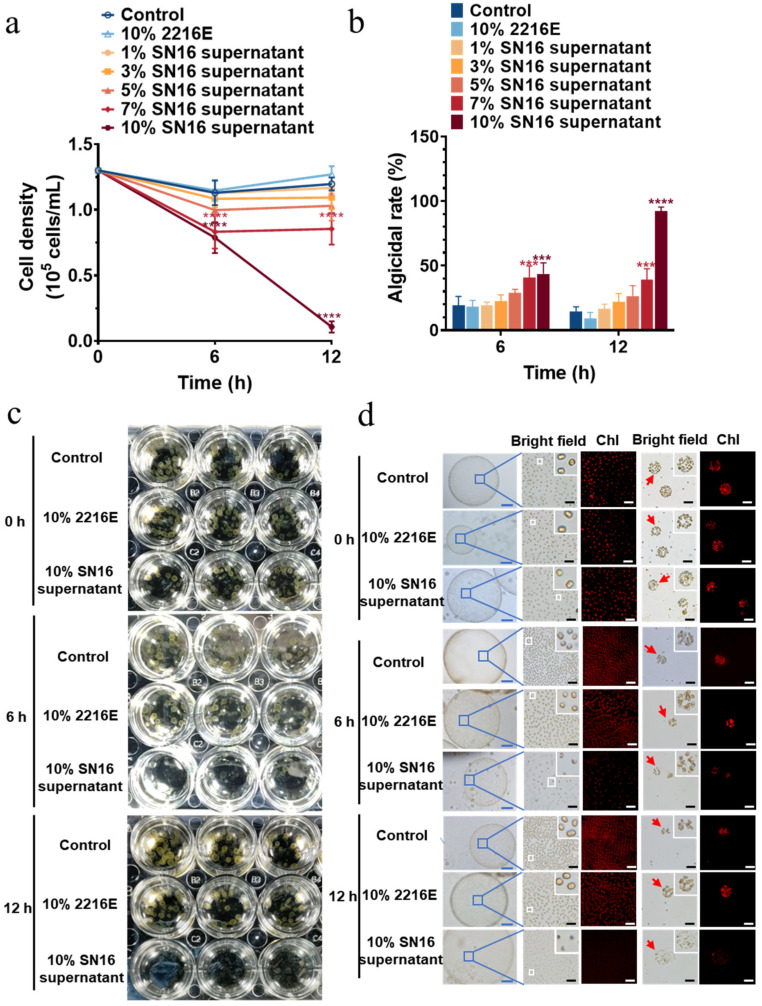
Algicidal activity of cell-free supernatant from strain SN16^T^ against colonies of *Pheaocystis globosa*. (**a**) Algicidal activities at various supernatant concentrations. *** and **** indicate the statistical significance *p* < 0.001 and *p* < 0.0001, respectively. (**b**) Corresponding algicidal rates calculated after 6 h and 12 h of co-incubation. *** and **** indicate the statistical significance *p* < 0.001 and *p* < 0.0001, respectively. (**c**) Stereomicrographs of algal colony treatments with the 10% supernatant; (**d**) Changes in chlorophyll autofluorescence of colonial cells after treatment with the 10% supernatant. Bar, 200 μm for blue and 40 μm for white and black. Chl, chlorophyll.

The SN16^T^ supernatant also exhibited notable algicidal activity against *P. globosa* colonies, although it was slightly less effective than against solitary cells at equivalent concentrations and time points (Figure 3a,b). After 12 h, the algicidal rates for colonies treated with the 7% and 10% supernatants were 39.6% and 90.3%, respectively. A microscopic examination of the colonies after 6 h of treatment revealed cellular vacuolation, indistinct cell boundaries, and diminished chlorophyll fluorescence. By 12 h, the colonies had turned white and lost their structural integrity, and chlorophyll fluorescence was nearly undetectable (Figure 3c,d). This suggests that the secreted algicidal compounds can penetrate the colonial matrix to kill the encapsulated cells.

### 3.3. Broad-Spectrum Algicidal Activity of Strain SN16^T^

To determine the algicidal spectrum of strain SN16^T^, its supernatant (10%, *v*/*v*) was tested against a panel of 15 microalgal species from 4 different phyla. As shown in Table 1, strain SN16^T^ demonstrated potent algicidal activity against a range of HAB-causing species. In addition to its effect on *P. globosa*, the supernatant effectively lysed six other dinoflagellate (Pyrrophyta) species, including *P. donghaiense*, *A. carterae*, *A. tamarense*, *A. catenella*, *K. mikimotoi*, and *S. trochoidea*. It also demonstrated activity against two diatom (Bacillariophyta) species, *T. weissflogii* and *S. costatum*. Conversely, no algicidal activity was observed against the tested species from Chlorophyta (*C. vulgaris*, *P. helgolandica*, and *N. oculate*) or Chrysophyta (*I. galbana*). These results suggest that SN16^T^ possesses a broad yet selective algicidal capacity, primarily targeting species within Pyrrophyta and Bacillariophyta, highlighting its potential for controlling diverse HABs. Although strain SN16^T^ demonstrates significant algicidal efficacy against various HAB algae, it has no discernible effect on *C. vulgaris*, *P. helgolandica*, and *N. oculata*, (Chlorophyta), which are economically important cultured green algae, nor on *I. galbana* (Haptopyta) which is used as fish feed.

### 3.4. Phylogenetic and Genomic Characterization of Strain SN16^T^

#### 3.4.1. 16 S rRNA Gene-Based Phylogenetic Analysis

**Figure 4:**
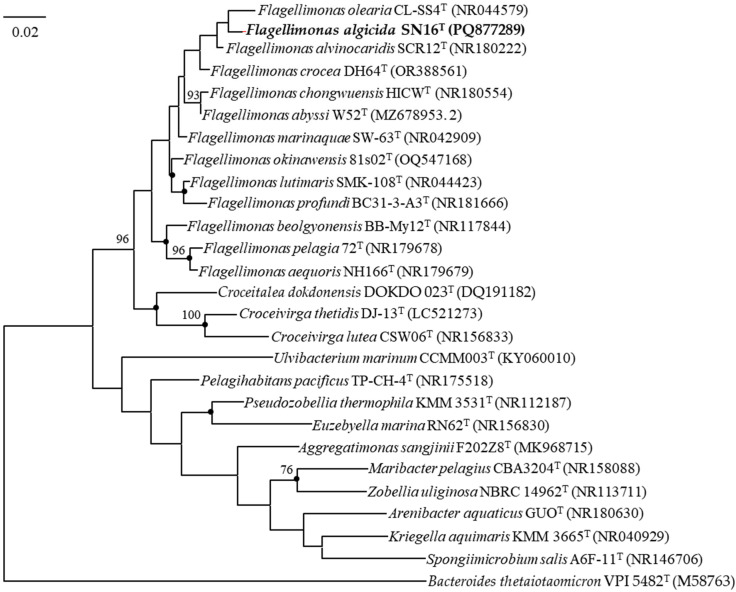
Maximum-likelihood phylogenetic tree based on 16S rRNA gene sequences, showing the phylogenetic position of SN16^T^ relative to those of other related taxa. The tree was constructed using the neighbor-joining (NJ) and minimum-evolution (ME) methods as well, and branches conserved across all three methods are indicated using filled circles. Bootstrap percentages (>70%) based on 1000 replications are shown at the branch points. *Bacteroides thetaiotaomicron* VPI 5482^T^ (M58763) was used as the outgroup. Bar, 0.02 substitutions per nucleotide position. The numbers in parentheses represent GenBank accession numbers of the 16S rRNA gene sequences.

Phylogenetic analyses based on the full-length 16S rRNA gene sequences (GenBank accession No. PQ877289) placed strain SN16^T^ within the genus *Flagellimonas* of the family *Flavobacteriaceae*. A sequence similarity analysis revealed its closest relatives to be *Flagellimonas alvinocaridis* SCR12^T^ (98.9% similarity), *Flagellimonas olearia* CL-SS4^T^ (98.5% similarity), *Flagellimonas crocea* DH64^T^ (98.1% similarity), and *Flagellimonas chongwuensis* HICW^T^ (97.8% similarity). In a phylogenetic tree constructed using the Maximum Likelihood method, strain SN16^T^ shared a common branch with its closest relative *F. olearia* CL-SS4^T^ and *F. alvinocaridis* SCR12^T^, thereby demonstrating its evolutionary position within the family *Flavobacteriaceae* (Figure 4). This phylogenetic placement was consistently supported by trees reconstructed using the NJ and ME algorithms (Figures S2 and S3).

#### 3.4.2. Genome Properties and Phylogenomic Analysis

The genome of strain SN16^T^ was sequenced. Contigs shorter than 1000 bp were removed, and the final assembled genome was deposited in the GenBank database under accession number JBMYIX000000000. The draft genome of strain SN16^T^ consists of 3,773,128 bp with a G + C content of 43.9%. This G + C content is distinct from those of its closest relatives: *F. olearia* CL-SS4^T^ (50.7%), *F. alvinocaridis* SCR12^T^ (42.3%), *F. crocea* DH64^T^ (42.6%), and *F. chongwuensis* HICW^T^ (41.4%). The genome annotation predicted 3481 protein-coding sequences (CDSs), 36 tRNA genes, and 5 rRNA genes (one 16S rRNA, one 23S rRNA, and three 5S rRNA).

**Figure 5:**
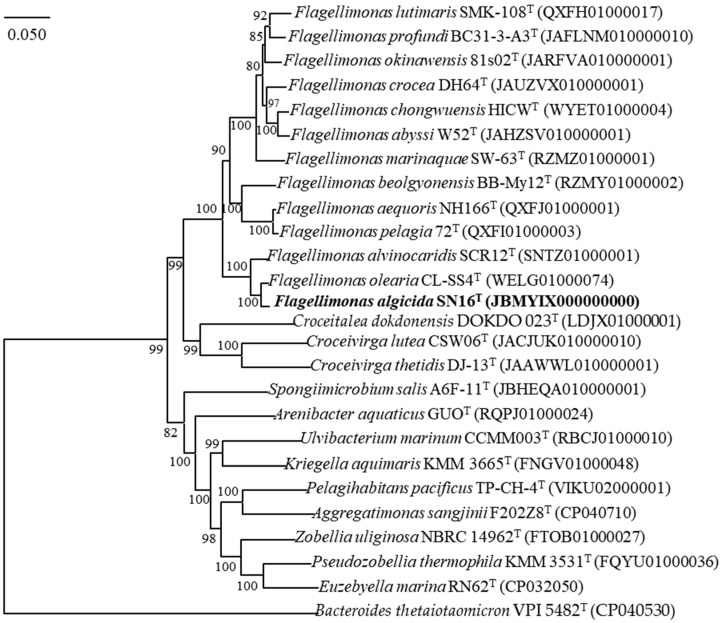
Whole-genome phylogenetic tree showing the phylogenetic position of strain SN16^T^ relative to those of other related taxa. Bootstrap percentage (>70%) based on 1000 replications are shown at the branch points. *Bacteroides thetaiotaomicron* VPI 5482^T^ was used as the outgroup. Bar, 0.05 substitutions per nucleotide position. The numbers in parentheses represent GenBank accession numbers of the genomes.

A whole-genome-based phylogenetic tree further clarified the relationship of strain SN16^T^ within the family *Flavobacteriaceae*, confirming its close affiliation with *F. olearia* CL-SS4^T^ and *F. alvinocaridis* SCR12^T^ (Figure 5). This result was consistent with the 16S rRNA gene-based phylogeny.

#### 3.4.3. Genomic Evidence for a Novel Species

To definitively determine the taxonomic status of strain SN16^T^, we calculated its ANI AAI and dDDH values against those of its closest relatives. The ANI and AAI values between strain SN16^T^ and its nearest neighbor, *F. olearia* CL-SS4^T^, were 91.37% and 94.8%, while those with *F. alvinocaridis* SCR12^T^ were 86.17% and 90.87%, respectively (Table 2). Both values fall below the 95–96% threshold generally accepted for bacterial species delineation. Furthermore, the dDDH values between strain SN16^T^ and *F. olearia* CL-SS4^T^ and *F. alvinocaridis* SCR12^T^ were 44.0% and 30.3%, respectively (Table 2). These values are significantly lower than the 70% threshold for conspecific strains [61,62].

ANI, AAI, and dDDH values that fall below the established species demarcation thresholds provide robust evidence that strain SN16^T^ represents a novel species within the genus *Flagellimonas*.

### 3.5. Physiological and Biochemical Characterization

Strain SN16^T^ exhibited optimal growth at 20–30 °C and pH 6.0–7.0. No growth was observed at temperatures below 15 °C or above 40 °C. The strain requires a minimum of 3% (*w*/*v*) NaCl for growth, with an optimal concentration of 4% and tolerance up to 8%. Its pH range for growth is 6.0–10.0. The temperature range is consistent with that for other members of the *Flagellimonas* genus, but its salinity and pH tolerance ranges are broader than those for its closest relatives: *F*. *olearia* CL-SS4^T^, *F*. *alvinocaridis* SCR12^T^, and *F*. *chongwuensis* HICW^T^ (Table 3).

Strain SN16^T^ tested positive for oxidase and catalase activity. It fermented D-glucose and utilized a range of substrates, including glucose, melibiose, saccharose (weakly), and amygdalin (weakly). It did not reduce nitrate to nitrite. The strain tested negative for the utilization of citrate, inositol, L-arabinose, D-mannose, D-mannitol, N-acetyl-glucosamine, D-maltose, potassium gluconate, capric acid, adipic acid, malic acid, trisodium citrate, and phenylacetic acid. An API ZYM analysis revealed positive results for alkaline phosphatase, esterase (C4), esterase lipase (C8), leucine arylamidase, valine arylamidase, cystine arylamidase, acid phosphatase, naphthol-AS-BI-phosphohydrolase, N-acetyl-β-glucosaminidase, and α-mannosidase (Table S1). Moreover, strain SN16^T^ showed negative results for lysine decarboxylase, ornithine decarboxylase, urease, tryptophane deaminase, and the utilization of citrate, as well as H_2_S and indole production. These common features suggest that strain SN16^T^ belongs to the genus *Flagellimonas*.

Key biochemical characteristics and substrate utilization patterns differentiating strain SN16^T^ from its phylogenetic neighbors are detailed in Table 3. Strain SN16^T^ exhibited a broader pH and NaCl growth range compared to its relatives. For instance, unlike *F. olearia* CL-SS4^T^, strain SN16^T^ exhibits activities for lipase (C14), trypsin, α-chymotrypsin, β-galactosidase (weakly), α-glucosidase, β-glucosidase and α-Mannosidase but tested negative for arginine dihydrolase, gelatinase, and gelatin hydrolysis. In contrast to other related species, it was positive for β-galactosidase (weakly) activities but negative for α-galactosidase and β-glucuronidase activities. It can utilize rhamnose and arabinose.

Regarding antibiotic susceptibility, strain SN16^T^ was sensitive to cefazonlin (30), cefoperazone (75), neomycin (30), kanamycin (30), streptomycin (10), and gentamicin (10). It was resistant to vancomycin (30), chloramphenicol (30), erythromycin (15), ampicillin (20), and novobiocin (30) (Table S2). These physiological and biochemical profiles share common features with genus *Flagellimonas*, while also presenting a unique combination of traits that distinguish it as a novel species.

### 3.6. Chemotaxonomic Characteristics

The chemotaxonomic profile of strain SN16^T^ is consistent with its classification within the genus *Flagellimonas*. The predominant isoprenoid quinone was identified as menaquinone-6 (MK-6). The major cellular fatty acids (>10%) were iso-C15:0 (42.15%) and iso-C15:1 G (27.97%) (Table 4). The polar lipid profile consisted of three unidentified polar lipids, one unidentified aminolipid, and one unidentified aminophospholipid (Figure S4).

### 3.7. Algicidal Activities and Assessment of the Secondary Metabolic Potential of the Genus Flagellimonas

**Figure 6:**
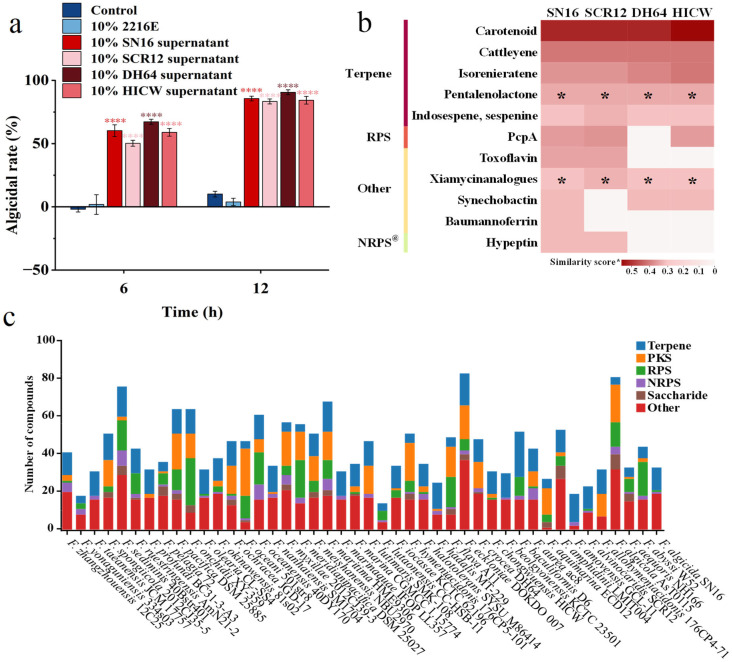
Comparative analysis of algicidal activity and secondary metabolite biosynthesis potential in *Flagellimonas* species. (**a**) Algicidal activities among four *Flagellimonas* species. **** indicates the the statistical significance *p* < 0.0001. (**b**) Secondary metabolite biosynthetic gene clusters (BGCs) identified in the genomes of four *Flagellimonas* species. The asterisk (*) indicates a strong correlation with algicidal activity. RPS: ribosomal peptide synthetases @: non-ribosomal peptide synthetases. (**c**) Secondary metabolite biosynthetic gene clusters (BGCs) identified in the genomes of 44 *Flagellimonas* species.

To determine whether algicidal activity is a conserved trait within the genus *Flagellimonas*, we analyzed the algicidal effects of culture supernatants from *F. alvinocaridis* SCR12^T^, *F. crocea* DH64^T^, and *F. chongwuensis* HICW^T^ against the harmful alga *P. globosa*. The results demonstrated that, consistent with strain SN16^T^, all three species exhibited potent algicidal effects, with algicidal rates exceeding 80% after 12 h of treatment. Among them, *F. crocea* DH64^T^ displayed the highest activity, reaching up to 91% (Figure 6a). These findings strongly suggest that multiple species within the *Flagellimonas* genus are capable of producing and secreting algicidal compounds.

Given that the algicidal activity of strain SN16^T^ was most pronounced during the stationary growth phase (Figure 1d), we hypothesized that the active compounds might be secondary metabolites. To investigate this possibility, we performed a comparative genomic analysis of all four strains using the anitSMASH platform. The analysis predicted a total of 11 known secondary metabolite biosynthetic gene clusters (BGCs) in the genome of strain SN16^T^, including those for terpenes, ribosomal peptide synthetase (RPS), non-ribosomal peptide synthetase (NRPS) products, and others (Figure 6b). Among these clusters, six metabolites were commonly predicted across all four algicidal strains: the terpenoids (carotenoid, cattleyene, isoreniseratene, pentalenolactone, and indosespene/sespenine) and xiamycin analogues classified under the “others” category.

Previous studies have reported that several *Flagellimonas* species can produce the antioxidant zeaxanthin, a dihydroxy derivative of β-carotene [36,37,38]. Consistent with these reports, we identified carotenoid BGCs in the genomes of all four strains, indicating that the ability to synthesize carotenoid derivatives may be a common trait within this genus. This finding explains the orange–yellow pigmentation observed in the strains during their stationary phase. However, given that algae also produce substantial quantities of carotenoids, isorenieratene and indosespene/sespenine, these compounds were ruled out as the primary algicidal agents. Therefore, we hypothesized that the algicidal activity is attributable to other shared metabolites, such as cattleyene, pentalenolactone, and xiamycin analogues. A comparative genomic analysis of 45 *Flagellimonas* species using antiSMASH revealed remarkable biosynthetic diversity and the capacity to produce structurally complex and pharmacologically relevant metabolites. Terpenes, RPSs, polyketide synthases (PKSs), and NRPSs were prevalent BGC types (Figure 6c), suggesting that algicidal activity may be a widespread trait across multiple species within this genus.

## 4. Discussion

### 4.1. Proposal of Flagellimonas Algicida sp. Nov.

Strain SN16^T^ shared the highest similarity with *F. alvinocaridis* SCR12^T^ (98.9%), followed by 98.5% with *F. olearia* CL-SS4^T^. Similarity to other species within the family *Flavobacteriaceae* was less than 95%. A phylogenetic analysis using the ML, NJ, and ME methods based on the 16S rRNA gene supported these relationships, further justifying the designation of strain SN16^T^ as a *Flagellimonas* strain within the family *Flavobacteriaceae*. Although the 16S rRNA gene sequence similarity to *F. alvinocaridis* SCR12^T^ (98.9%) was higher than the 98.65% threshold for species delineation, the overall genome relatedness indices, including ANI, AAI, and dDDH values, between SN16^T^ and other *Flagellimonas* type strains were below the proposed species thresholds of 95.0–96.0% and 70.0%, respectively [61]. Based on a maximum-likelihood phylogenomic tree constructed using the EzBioCloud database, strain SN16^T^ clustered with *F. olearia* CL-SS4^T^ and *F. alvinocaridis* SCR12^T^ but was distinct from other *Flagellimonas* type strains. Collectively, the distinct phylogenetic position, coupled with the ANI, AAI, and dDDH values below the established species demarcation thresholds, provides robust evidence that strain SN16^T^ represents a novel species within the genus *Flagellimonas*. Strain SN16^T^ could be differentiated from *F. olearia* CL-SS4^T^ and *F. alvinocaridis* SCR12^T^ by its growth characteristics (e.g., NaCl concentrations and optimal temperatures), enzyme activities (including hydrolysis of gelatin, lipase (C14), trypsin, α- and β-galactosidases, α- and β-glucosidase, α-mannosidase, and gelatinase), sole carbon source utilization (D-mannitol), acid production from D-glucose and D-mannose, DNA G + C content, and cellular fatty acid composition. Based on these combined genetic, genomic, phylogenomic, and chemotaxonomic characteristics, strain SN16^T^ is identified as a novel *Flagellimonas* species, for which the name *Flagellimonas algicida* sp. nov. is proposed.

### 4.2. Description of Flagellimonas algicida sp. Nov.

*Flagellimonas algicida* sp. nov. (etymology: al.gi′ci.da. L. fem. n. *alga -ae* alga; L. suff. -*cida* from L. v. *caedere* to cut or to kill; N.L. n. *algicida* related to alga-killer, referring to the strain’s potent algicidal activity).

Its cells are Gram-stain-negative, non-flagellated, long rod-shaped (1.84 × 0.34 μm) during the logarithmic phase, and transitions to a coccoid form (0.58 μm in diameter) during the stationary phase. Colonies on ZoBell 2216E agar are round, light-yellow, and smooth and have regular edges. Growth occurs at 15–40 °C (optimum, 20–30 °C) and pH 6–10 (optimum, pH 6–7) and in the presence of 3–8% (*w*/*v*) NaCl (optimum, 4%). The strain is positive for oxidase and catalase. The major fatty acids are iso-C15:0 and iso-C15:1 G. The major respiratory lipoquinone is MK-6. The predominant polar lipids consist of three unidentified polar lipids, one unidentified aminolipid, and one unidentified aminophospholipid. The strain can utilize glucose and melibiose and shows weak utilization for sorbitol, saccharose, mannitol, and amygdalin. It can ferment D-glucose and produce acetoin. Nitrate is not reduced to nitrite. The strain is positive for the enzyme activities of lipase (C14), trypsin, α-chymotrypsin, α-glucosidase, β-glucosidase, and α-mannosidase. The DNA G + C content calculated from the genome sequence is 43.9%. The genome size is approximately 3.8 Mb and harbors biosynthetic gene clusters encoding terpenes, ribosomal peptides, non-ribosomal peptides, and others.

The type strain, SN16^T^ (=MCCC 1K09924^T^ = KCTC 102450^T^), was isolated from surface seawater samples collected in December 2023 from Dadeng Island (Xiamen, China) during a *P. globosa* bloom. The NCBI GenBank accession number for the 16S rRNA gene and genome sequence of strain SN16^T^ are PQ877289 and JBMYIX000000000, respectively.

### 4.3. Algicidal Property of Flagellimonas algicida sp. Nov.

Outbreaks of *P. globosa* seriously threaten aquaculture and coastal nuclear power safety and causes economic losses. Therefore, novel algal removal technologies are urgently needed to control *P. globosa* blooms. Although a few algicidal bacteria have shown high algal-lytic activity against solitary cells of *P. globosa* [10,11,12,13,14,15,17,18], their effectiveness against the colonial form of *P. globosa* is limited. Strain SN16^T^ exhibits strong algicidal activity against the colonial form of *P. globosa*, suggesting its promising potential as a biocontrol agent. To determine its host specificity, we evaluated its algicidal activity against eleven other common HAB species. This panel included six dinoflagellate (Pyrrophyta) species, four diatom (Bacillariophyta) species, and *H. akashiwo* from the division Xanthophyta. The results demonstrated significant algicidal activity against all tested HAB species, indicating its potential for the mitigation of diverse HAB events. To assess potential harm to beneficial algae, we conducted algicidal assays on three economically important mariculture species from the division Chlorophyta and *I. galbana*—a common fish feed alga from the division Chrysophyta. Strain SN16^T^ demonstrated no algicidal activity against the tested mariculture green algae and the fish feed alga. This suggests favorable safety and minimal risk of negative impact on alga aquaculture. However, ecological safety evaluations are needed prior to the application of strain SN16^T^ for controlling HABs. An integrated approach to ecotoxicological assessment, incorporating a range of representative aquatic species (e.g., bacteria, algae, invertebrates, and fish), is crucial for accurately predicting the risk posed by effluents or toxic chemicals to aquatic biota.

Although 45 *Flagellimonas* species have been identified, their ecological roles and potential applications are largely unknown. At present, the known functions of *Flagellimonas* species are limited to the production of the antioxidant zeaxanthin, the degradation of AHLs, and the enzymatic degradation of fucoidan. This study demonstrates, for the first time, that four *Flagellimonas* species produce algicidal metabolites, suggesting their potential for development as biocontrol agents for HAB management. We identified specific BGCs (pentalenolactone and xiamycin analogues) that are uniquely shared among four potent algicidal strains. This shared genomic feature strongly correlates with the shared algicidal phenotype. Therefore, we predicted that terpenoids (pentalenolactone) and xiamycin analogues are highly plausible candidates through a comparative genomic analysis of all four strains with algicidal capacity using the antiSMASH platform. Future work will focus on the isolation, purification, and structural elucidation of these candidate compounds from *Flagellimonas* species to confirm their algicidal activity and assess their environmental persistence and degradation pathways. Furthermore, the comparative genomic analysis of 45 *Flagellimonas* species revealed remarkable biosynthetic diversity and the capacity to produce structurally complex and pharmacologically relevant metabolites, highlighting the promising potential of this genus in biotechnology and pharmaceuticals.

## 5. Conclusions

In this study, we characterized a novel bacterial strain, SN16^T^, isolated from a harmful algal bloom. Polyphasic evidence from genomic, chemotaxonomic, and physiological analyses identified strain SN16^T^ as a novel species within the genus *Flagellimonas*. We propose the name *Flagellimonas algicida* sp. nov. for this species.

A key finding of this study is the potent and broad-spectrum algicidal activity of this new species. Strain SN16^T^ effectively lyses nine microalgal species responsible for HABs, encompassing various dinoflagellates and diatoms. To the best of our knowledge, this is the first report of any *Flagellimonas* species exhibiting broad-spectrum algicidal activity, notably including efficacy against the colonial form of *P. globosa*—a key ecological challenge in HAB mitigation. Therefore, the discovery of strain SN16^T^ not only expands the known diversity of the family *Flavobacteriaceae* but, more significantly, also identifies a highly promising candidate for the development of novel biocontrol agents to mitigate harmful algal blooms.

Furthermore, this algicidal ability was subsequently demonstrated to be a common characteristic among three other *Flagellimonas* members. This finding suggests that the production of algicidal compounds might be a conserved trait across multiple species within the genus, highlighting its potential for broader biotechnological applications in combating HABs. Future research will focus on isolating and characterizing these active algicidal compounds, as well as exploring the ecological roles and mechanisms underlying this unique activity.

## Figures and Tables

**Table 1:** Algicidal activities of 10% SN16^T^ supernatant for various alga species.

Phylum	Species	Algicidal Activity (%)
Chlorophyta	*Chlorella vulgaris*	-
	*Tetraselmis helgolandica*	-
	*Nannochloris oculata*	-
Pyrrophyta	*Prorocentrum donghaiense*	73.48
	*Amphidinium carterae*	93.01
	*Alexandrium tamarense*	93.17
	*Alexandrium catenella*	84.09
	*Karenia mikimotoi*	89.01
	*Scrippsiella trochoidea*	77.67
Bacillariophyta	*Entomoneis alata*	-
	*Conticribra weissflogii*	87.19
	*Skeletonema costatum*	59.09
	*Cylindrotheca closterium*	-
Chrysophyta	*Isochrysis galbana*	-
Haptophyta	*Phaeocystis globosa*	96.8%
Xanthophyta	*Heterosigma akashiwo*	-

**Table 2:** Summary of average nucleotide identity (ANI), average amino acid identity (AAI), and digital DNA–DNA hybridization (dDDH) values between strain SN16^T^ and nine type strains within the genus *Flagellimonas*.

	Strains	GenBank Accession Number	ANI (%)	AAI (%)	dDDH (%)
1	*Flagellimonas olearia* CL-SS4^T^	WELG01000074	91.37	94.80	44.0
2	*Flagellimonas alvinocaridis* SCR12^T^	SNTZ01000001	86.17	90.87	30.3
3	*Flagellimonas beolgyonensis* KCTC 23501^T^	RZMY01000002	76.18	80.21	19.3
4	*Flagellimonas yonaguniensis* 334s03^T^	JARFVB010000001	76.00	80.08	19.4
5	*Flagellimonas ruestringensis* DSM 13258^T^	CP002999	75.76	79.85	19.3
6	*Flagellimonas oceani* 501str8^T^	CP049616	75.56	79.21	19.0
7	*Flagellimonas aurea* ac8^T^	CP159476	75.48	79.31	18.9
8	*Flagellimonas chongwuensis* HICW^T^	WYET01000004	75.47	79.47	18.9
9	*Flagellimonas crocea* DH64^T^	JAUZVX010000001	75.38	79.67	19.3

**Table 3:** Comparative analysis of phenotypic characteristics distinguishing strain SN16^T^ from closely related species.

Characteristic	1	2 *	3	4	5
Temperature range for growth (°C) (Optimum)	15–40 (20–30)	15–40 (25–30)	16–40 (37) ^@^	10–40 ^#^	15–40 (25–30) ^&^
NaCl range for growth (%, *w*/*v*) (Optimum)	3–8 (4)	1–6(2–3)	1–5 (3) ^@^	0.5–8.0 ^#^	0.5–8 (2–3) ^&^
pH range for growth (Optimum)	6–10 (6–7)	5.2–9.4 (6.8–7.7)	5.5–8.5 (6.5) ^@^	5.5–8.5^#^	6–8 (7) ^&^
Oxidase	+	+	− ^@^	+	− ^&^
Catalase	+	+	− ^@^	+	+ ^&^
Enzyme activity (API ZYM)					
Lipase (C14)	+	−	w	+	+
Trypsin	+	−	+	+	+
α-Chymotrypsin	+	−	+	+	+
α-Galactosidase	−	−	+	+	+
β-Galactosidase	w	−	+	+	+
β-Glucuronidase	−	−	+	−	−
α-Glucosidase	+	−	+	+	+
β-Glucosidase	+	−	+	+	+
α-Mannosidase	+	−	+	+	+
α-Fucosidase	−	−	+	−	−
API 20E results:					
Arginine dihydrolase	−	w	−	−	−
Gelatinase	−	+	−	−	−
Mannitol	w	−	w	w	−
Rhamnose	−	NM	w	w	−
Saccharose/Amygdalin	w	NM	+	w	+
Arabinose	−	−	−	w	+
API 20NE results:					
Reduction of nitrate to nitrite	−	−	+	−	w
Denitrification	−	NM	+	−	w
D-Glucose fermentation	+	NM	+	w	+
Gelatin hydrolysis	−	+	−	−	−
D-Glucose	−	+	−	−	−
D-Mannose	−	+	−	−	−
Major respiratory quinone	MK−6	MK−6	MK−6 ^@^	MK−6 ^#^	MK−6 ^&^
DNA G + C content (mol%)	43.9	50.7	42.3 ^@^	42.6 ^#^	41.4 ^&^

Taxa: 1, *Flagellimonas algicida* SN16^T^; 2, *Flagellimonas olearia* CL-SS4^T^; 3, *Flagellimonas alvinocaridis* SCR12^T^; 4, *Flagellimonas crocea* DH64^T^; 5, *Flagellimonas chongwuensis* HICW^T^. +, positive; w, weakly positive reaction; −, negative; NM, not mentioned in the references. * Data from Chung Y. Hwang et al. [63]. @ Data from Lijun Liu et al. [29]. # Data from Minglei Wang et al. [64]. & Data from Mingxia Chen et al. [31].

**Table 4:** Cellular fatty acid composition (%) of strain SN16^T^ and recognized *Flagellimonas* species.

	1	2 *	3	4	5
Straight-chain					
C_15:0_	−	10.7	−	−	−
C_16:0_	1.09	TR	1.46	1.36	TR
C_18:0_	−	−	2.81	TR	TR
Branched					
iso-C_13:0_	1.19	−	TR	1.17	1.37
iso-C_15:0_	42.15	18.7	38.55	49.06	46.43
anteiso-C_15:0_	2.69	1.4	TR	3.12	1.59
iso-C_15:1_ G	27.97	17.1	21.28	24.33	28.76
iso-C_16:0_	TR	TR	TR	1.83	TR
iso-C_17:1_ω9c	−	3.6	−	−	−
Unsaturated					
C_15:1_ω6c	TR	1.4	TR	TR	TR
C_17:1_ω6c	TR	1.6	1.34	TR	TR
C_20:2_ω6,9c	TR	−	TR	1.10	TR
Hydroxy					
C_15:0_ 3-OH	TR	1.9	−	4.19	TR
iso-C_15:0_ 3-OH	5.93	4.4	4.83	−	5.39
iso-C_16:0_ 3-OH	TR	1.4	TR	TR	TR
C_17:0_ 3-OH	TR	1.2	TR	TR	TR
iso-C_17:0_ 3-OH	4.52	20.5	4.78	4.06	6.01
Summed Features ^#^					
1	−	7.1	−	−	−
3	4.68	−	−	1.30	3.23
8	TR	−	−	TR	TR
9	2.88	−	−	2.38	TR

Taxa: 1, *Flagellimonas algicida* SN16^T^; 2, *Flagellimonas olearia* CL-SS4^T^; 3, *Flagellimonas alvinocaridis* SCR12^T^; 4, *Flagellimonas crocea* DH64^T^; 5, *Flagellimonas chongwuensis* HICW^T^. Fatty acids, representing less than 1.0% in all strains, were omitted. TR: traces (<1.0%); −, not detected/not reported. * Data from Chung Y. Hwang et al. [63]. # Summed feature 1 contains C_16:1_ω7c and/or iso-C_15:0_ 2-OH; summed feature 3 contains C_16:1_ω6c and/or C_16:1_ω7c; summed feature 8 contains C_18:1_ω6c or C_18:1_ω7c; summed feature 9 contains 10-methyl C_16:0_ or iso-C_17:1_ω9c.

## Data Availability

Whole-genome sequencing data of the new strain *Flagellimonas algicida* SN16^T^ has been deposited at GenBank under the accession number JBMYIX000000000. Its 16S rRNA gene sequence has been deposited under the accession number PQ877289.

## Supplementary Materials

The following supporting information can be downloaded at https://www.mdpi.com/article/10.3390/microorganisms13092062/s1, Figure S1: Schematic workflow of the experimental design. Figure S2: Neighbor-joining phylogenetic tree based on the 16S rRNA gene sequences showing phylogenetic relationships of strain SN16^T^ and representatives of other related taxa. Figure S3: Minimum-evolution phylogenetic tree based on 16S rRNA gene sequences showing the phylogenetic positions of SN16^T^ and representatives of other related taxa. Figure S4: Two-dimensional thin-layer chromatogram of total polar lipids from strain SN16^T^. Table S1: Physiochemical characteristics of strain SN16^T^ consistent with closely related species. Table S2: Antibiotic susceptibility of strain SN16^T^.
