# Supplementary material for: Flagellimonas algicida sp. Nov.: A Novel Broad-Spectrum Algicidal Bacterium Targeting Harmful Algal Bloom Species and Genomic Insights into Its Secondary Metabolites
Source: Microorganisms. 2025 Sep 4;13(9):2062. doi: 10.3390/microorganisms13092062 (PMC12472895; doi:10.3390/microorganisms13092062)
Supplement: Supplementary file 1 [file microorganisms-13-02062-s001.zip › microorganisms-3789314-supplementary.pdf]

Supplementary materials

Figure S1

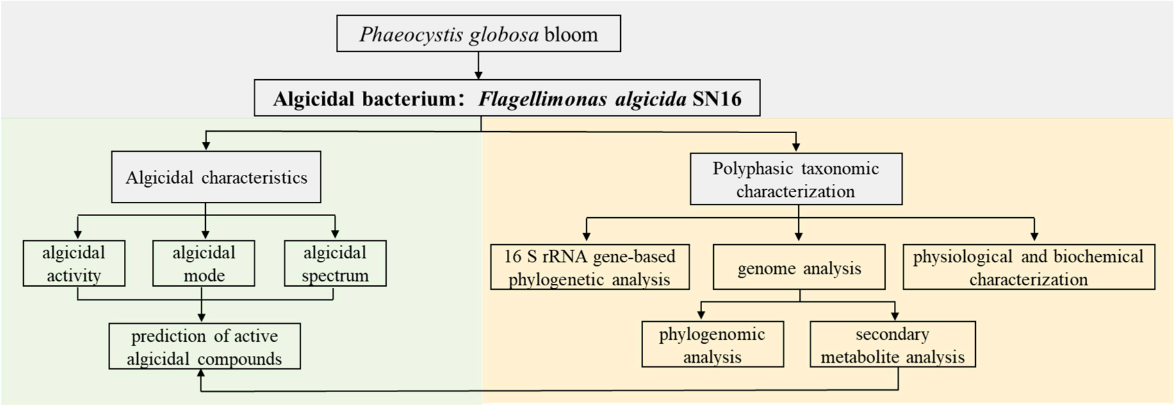

Figure S1. Schematic workflow of the experimental design.

Figure S2

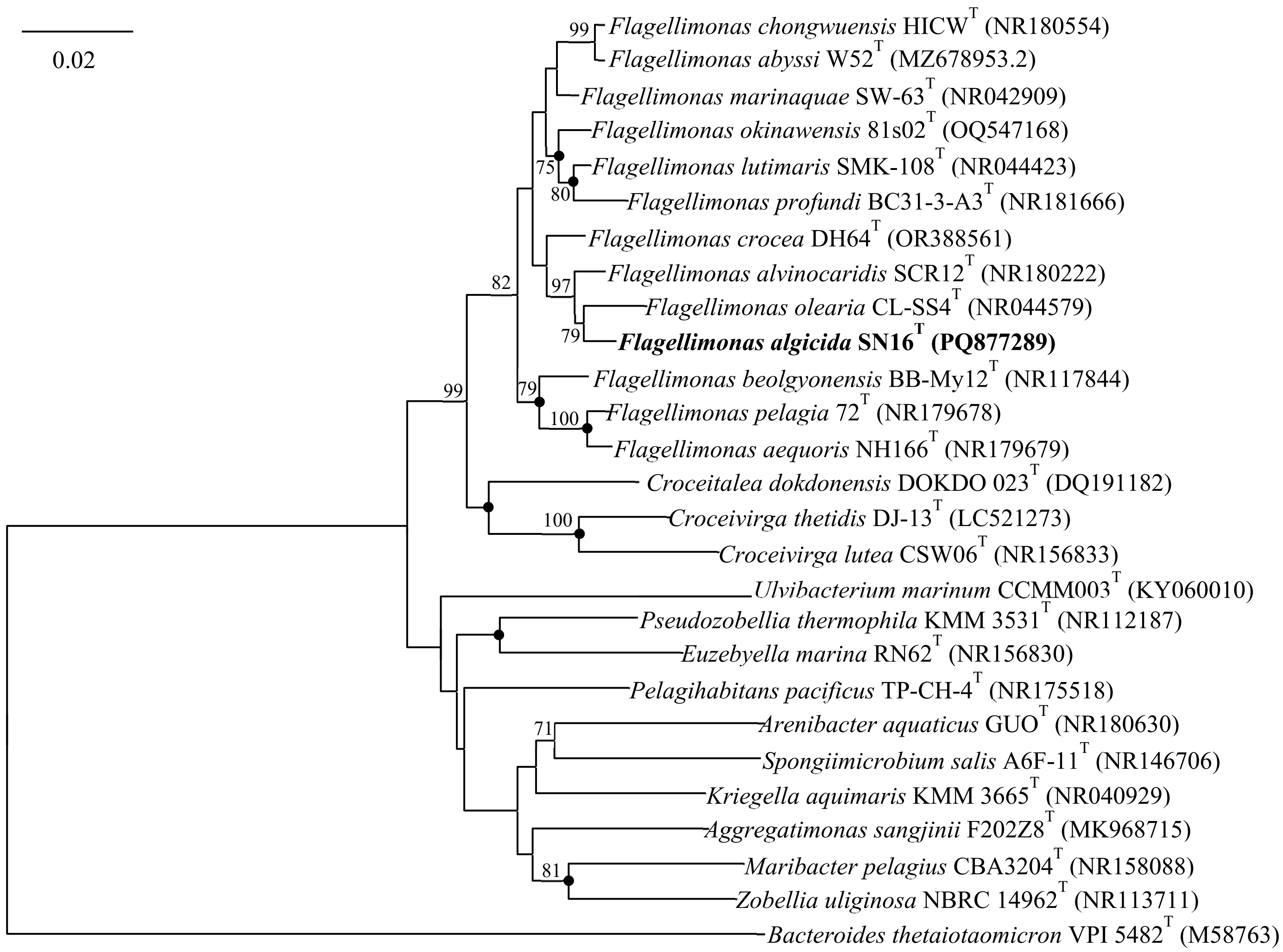

Figure S2. Neighbor-joining phylogenetic tree based on 16S rRNA gene sequences, showing the phylogenetic positions of SN16^{T} and representatives of other related taxa. The filled circles indicate branches of the tree that were also formed using the minimum-evolution (ME) and maximum-likelihood (ML) method. Bootstrap percentages (>70%) based on 1000 replications are shown at the branch points. *Bacteroides thetaiotaomicron* VPI 5482^{T} (M58763) as used as the outgroup. Bar, 0.02 substitutions per nucleotide position.

Figure S3

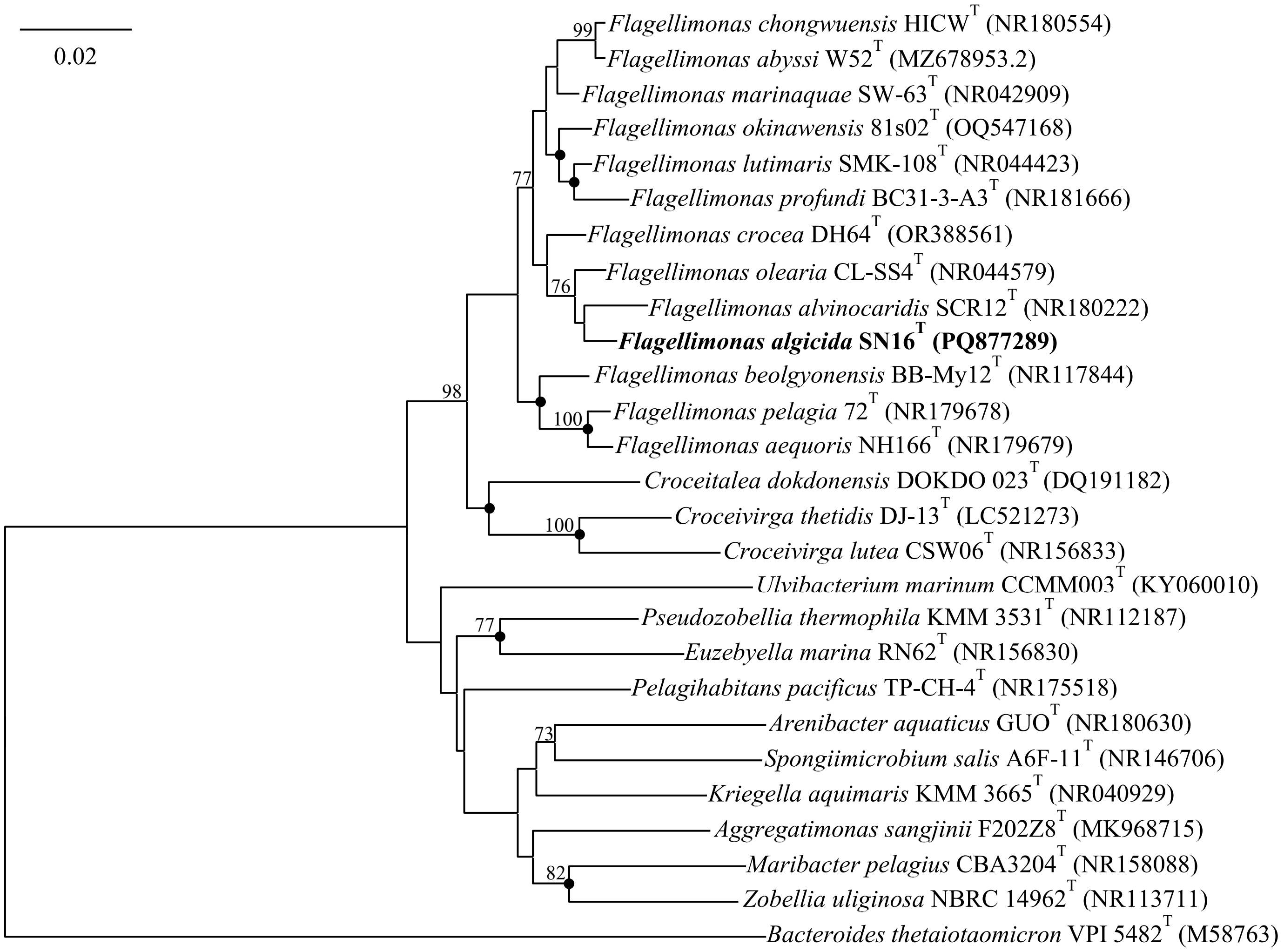

Figure S3. Minimum-evolution phylogenetic tree based on 16S rRNA gene sequences showing the phylogenetic positions of SN16^T and representatives of other related taxa. The filled circles indicate branches of the tree that were also formed using the neighbor-joining (NJ) and maximum-likelihood (ML) method. Bootstrap percentages (>70%) based on 1000 replications are shown at the branch points. *Bacteroides thetaiotaomicron* VPI 5482^T (M58763) was used as the outgroup. Bar, 0.02 substitutions per nucleotide position.

Figure S4

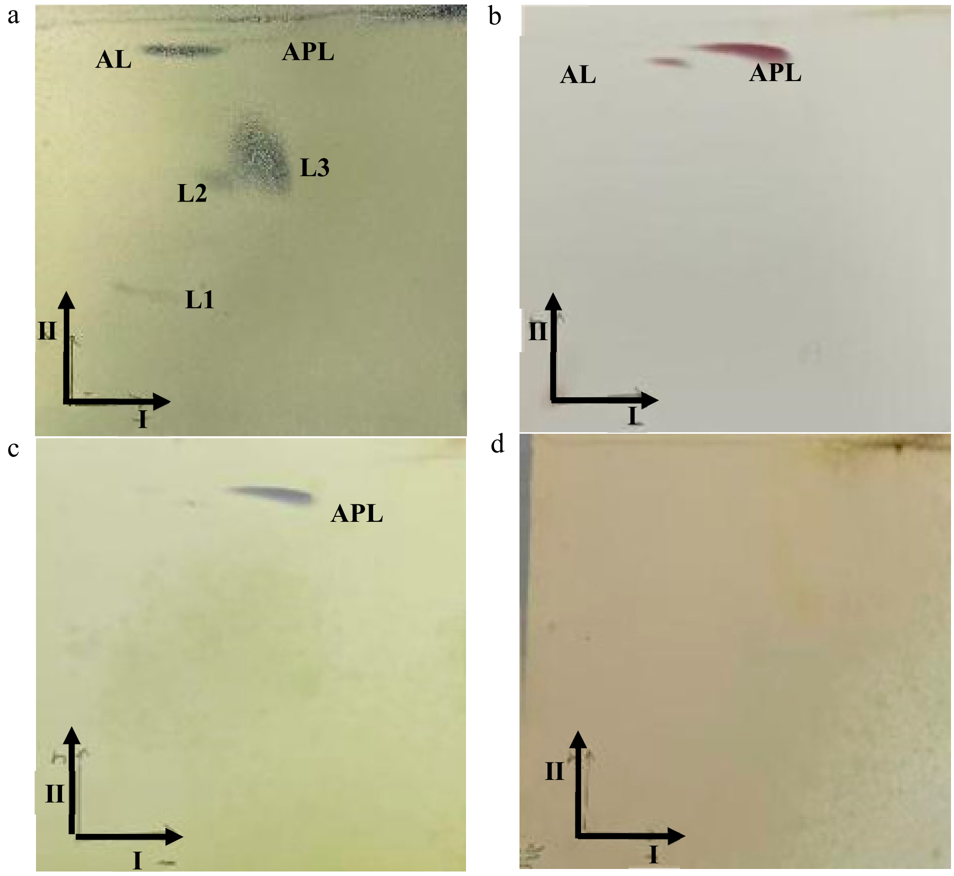

Figure S4. Two-dimensional thin-layer chromatogram of total polar lipids from strain SN16^T.

a, molybdphosphoric acid; b, ninhydrin; c, molybdenum blue; d, 1-naphthol sulfate ethanol. L1-L3, unknown polar lipids; AL, unknown aminolipid; APL, unknown aminophospholipid.

Table S1 Physiochemical characteristics of strain SN16^T consistent with closely related species.

Taxa: 1, SN16^T; 2, *Flagellimonas olearia* CL-SS4^T; 3, *Flagellimonas alvinocaridis* SCR12^T; 4, *Flagellimonas crocea* DH64^T; 5, *Flagellimonas chongwuensis* HICW^T. +, positive; w, weakly positive reaction; −, negative; NM, not mentioned in the references.

Characteristic	1	2*	3	4	5
Enzyme activity (API ZYM)					
Alkaline phosphatase	+	+	+	+	+
Esterase(C4)	+	+	+	+	+
Esterase lipase (C8)	+	+	+	+	+
Leucine arylamidase	+	+	+	+	+
Valine arylamidase	+	+	+	+	+
Cystine arylamidase	+	+	+	+	+
Acid phosphatase	+	+	+	+	+
Naphtol-AS-BI-phosphohydrolase	+	+	+	+	+
N-Acetyl-β-glucosaminidase	+	+	+	+	+
API 20E results:					
Lysine decarboxylase	−	NM	−	−	−
Ornithine decarboxylase	−	NM	−	−	−
Citrate utilization	−	−	−	−	−
H_2S production	−	−	−	−	−
Urease	−	−	−	−	−
Tryptophane deaminase	−	NM	−	−	−
Indole production	−	NM	−	−	−
Voges Proskauer	+	NM	+	+	+
Glucose	+	+	+	+	+
Inositol	−	−	−	−	−
Melibiose	+	NM	+	+	+
API 20NE results:					
L-Arabinose	−	−	−	−	−
D-Mannitol	−	−	−	−	−
N-Acetyl-glucosamine	−	NM	−	−	−
D-Maltose	−	NM	−	−	−
Potassium gluconate	−	NM	−	−	−
Capric acid	−	NM	−	−	−
Adipic acid	−	NM	−	−	−
Malic acid	−	NM	−	−	−
Trisodium citrate	−	NM	−	−	−
Phenylacetic acid	−	NM	−	−	−

* Data from Chung Y. Hwang et al.

Table S2 Antibiotic susceptibility of strain SN16^T.

Antibiotic	susceptibility
chloramphenicol (30)	+
vancomycin (30)	+
kanamycin (30)	−
cefazoline (30)	−
streptomycin (10)	−
penicillin (10 U)	+
cefoperazone (75)	−
neomycin (30)	−
erythromycin (15)	+
gentamicin (10)	−
ampicillin (20)	+
novobiocin (30)	+

Taxa: +, sensitive; −, resistant.
